# Identification of Novel Copy Number Variations of VCAN Gene in Three Chinese Families with Wagner Disease

**DOI:** 10.3390/genes11090992

**Published:** 2020-08-25

**Authors:** Songshan Li, Mengke Li, Limei Sun, Xiujuan Zhao, Ting Zhang, Li Huang, Sijian Huang, Chonglin Chen, Zhirong Wang, Xiaoyan Ding

**Affiliations:** State Key Laboratory of Ophthalmology, Pediatric Department, Zhongshan Ophthalmic Center, Sun Yat-sen University, Guangzhou 510000, China; lisongshan@gzzoc.com (S.L.); limengke@gzzoc.com (M.L.); sunlimei@gzzoc.com (L.S.); zhaoxiujuan@gzzoc.com (X.Z.); zhangting@gzzoc.com (T.Z.); huangli@gzzoc.com (L.H.); huangsijian@gzzoc.com (S.H.); chenchonglin@gzzoc.com (C.C.); wangzhitong@gzzoc.com (Z.W.)

**Keywords:** Wagner disease, VCAN, versican, copy number variations, CNVs

## Abstract

The VCAN/versican gene encodes an important component of the extracellular matrix, the chondroitin sulfate proteoglycan 2 (CSPG2/versican). Heterozygous variants targeting exon 8 of VCAN have been shown to cause Wagner disease, a rare autosomal dominant non-syndromic vitreoretinopathy that induces retinal detachment, cataracts and permanent visual loss. In this study, we report on six patients from three unrelated families with Wagner disease in whom we identified three novel copy number variations of VCAN. Quantitative real-time polymerase chain reaction analysis identified deletions, including one exon–intron boundary of exon 8 or both exons 8 and 9, causing the haploinsufficiency of VCAN mRNAs.

## 1. Introduction

Wagner disease (WD) (MIM #143,200) is a rare hereditary non-syndromic autosomal dominant vitreoretinopathy characterized by optically empty vitreous and avascular vitreous strands and veils [1]. Additional ocular clinical manifestations include mild to moderate myopia, cataracts, chorioretinal atrophy with progression, retinal pigmentation, ectopic foveae and retinal detachment [2,3,4,5,6]. Typically, the symptoms of WD manifest during adolescence but can start in early childhood. By contrast with Stickler syndrome, systemic abnormalities are usually not present in WD [7].

The disease-causing molecular defect for WD is a mutation of the VCAN/versican gene [3,8]. This gene is located on chromosome 5q13-q14 and consists of 15 exons which encode chondroitin sulfate proteoglycan 2 (CSPG2/versican). Among the components of the extracellular matrix, CSPG2/versican is involved in the maintenance of the vitreous body in the human eye. The central area of CSPG2/versican is encoded by two large exons (exons 7 and 8), yielding four physiological isoforms (V0, V1, V2 and V3) by alternative splicing [3,4,5,9,10].

To date, 11 different VCAN single nucleotide variants, all located on the conserved exon 8 splice site, have been identified in 20 families with Wagner syndrome using systematic sequence analysis on either side of the intron–exon boundary of exon 8 by Sanger sequencing or next-generation sequencing (NGS). In addition, three deletions encompassing exon 8 and only exon 8 of VCAN, yielding copy number variations (CNVs) of exon 8 amplicons, were detected using NGS accompanied by CNV analysis (Table 1). Despite the fact that VCAN deletion and single-nucleotide changes are distinct molecular defects established in Wagner patients, both changes specifically target exon 8 and elicit the splicing mutation of VCAN. These mutations lead to the aberrant expression of the four VCAN isoforms with increased V2 and V3 isoforms and haploinsufficiency of V0 and V1 isoforms. Based on these findings, imbalanced versican isoforms were considered as the possible molecular underpinnings of Wagner syndrome.

Currently, the single nucleotide variants in the splicing site of exon 8 are still the primary molecular defects identified. Nevertheless, a previous study of three WD families found no VCAN splice site mutations; these cases were found to have CNVs overlapping VCAN exon 8 using a targeted-NGS approach [11,12]. Analogous to these cases, we identified three novel CNVs of VCAN in three unrelated Chinese families with WD, who also had no detectable splice site mutations. Here, we present the extensive study of these Wagner cases and analyze the clinical phenotypes associated with these CNV mutations.

## 2. Materials and Methods

This study was conducted adhering to the Declaration of Helsinki and was approved by the institutional review boards of Zhongshan Ophthalmic Center (ZOC) of Sun-Yat-Sen University (2014MEKY048). Written informed consent was obtained from the participants or guardians of children under 18 years of age.

The WD probands were recruited from January 2018 to March 2020 in the pediatric retinal disease clinic. The clinical diagnosis of WD was based on typical clinical manifestations. All probands were born full-term. In total, 6 WD patients (XDW894 from kindred A; XDW789 and XDW790 from kindred B; XDW286, XDW288 and XDW289 from kindred C) and three unaffected normal control or family members (HSR863 as normal control for kindred A, XDW905 from kindred B, XDW287 from kindred C) were included from 3 families. A comprehensive ophthalmic examination was performed on all family members, including best corrected visual acuity, slit-lamp biomicroscopy, intraocular pressure measurement, fundus examination, fundus photography, full-field electroretinography and spectral-domain optical coherence tomography. Blood samples were collected for DNA extraction and genetic analysis. Whole-exome sequencing (WES) and copy number variations analysis were performed in the probands and their parents. In the exome sequencing, the submitted regions included all coding regions, their intron boundaries (±25 bp) and all known splice sites. The Human Gene Mutation Database, Genome Aggregation Database and Exome Aggregation Consortium were used to identify the reported pathogenic variants. The seqCNV was used for the CNV analysis based on the sequencing depth, with the unaffected individuals as normal control (HSR863 as normal control for kindred A, XDW905 for kindred B and XDW287 for kindred C) [13].

The mRNA was extracted from fresh peripheral blood cells with the Blood-to-CT nucleic acid preparation kit (Thermo Fisher, Waltham, MA, USA). Quantitative real-time polymerase chain reactions (qRT-PCR) on the VCAN gene, with primers specific to the presumed deletion (Supplementary Table S1), were performed for the CNV validations with SDHA as an internal control [12]. For each qRT-PCR reaction, 5 ng DNA, 2 µmol/L of forward/reversed primer and 15 µL SYBR Green Master Mix (Life Technologies, Grand Island, NY, USA) were used. A standard thermocycling program was used for the amplification by the qPCR system (ABI StepOnePlus, Foster City, CA, USA). For each sample, at least three PCR reactions were performed to ensure reproducibility. The differences in expression between patients and controls were calculated by using the 2^ΔΔCp^ method [17]. Student’s *t*-test was used for the statistical analysis of the qPCR data by GraphPad (GraphPad Software, San Diego, CA, USA). The level of significance was set as *p* = 0.05.

## 3. Results

### 3.1. Ocular Phenotypes

**Figure 1:**
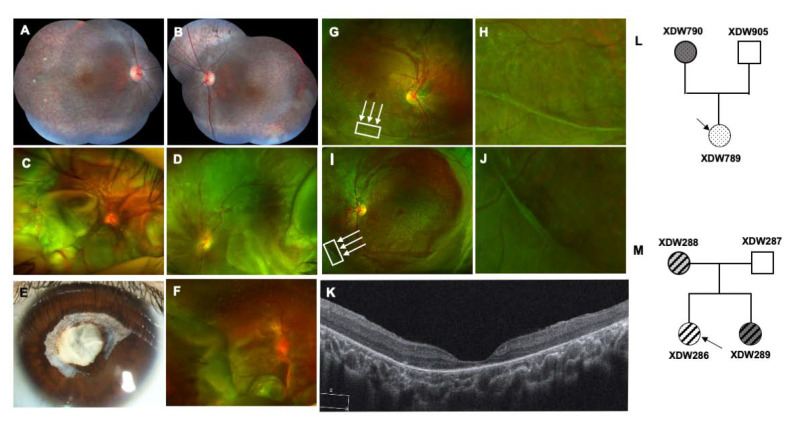
Ocular findings for the probands in three families with Wagner disease (WD): (**A**–**D**): Ocular imaging in kindred A; mosaic multifield fundus photographs of OD (**A**) and OS (**B**) show diffuse pigmentation with chorioretinal atrophy and retinal pigmentary degeneration. Ultra-wide-field scanning laser ophthalmoscopic images on his second visit demonstrate retinal detachment of both eyes (**C**,**D**). (**E**,**F**): Ocular imaging in kindred B; pupillary synechiae and cataracts are detected in the left eye (**E**). The fundus photograph of the proband’s right eye (**F**) shows retinal detachment in her right eye. (**G**–**K**): Ocular imaging in kindred C; ultra-wide-field scanning laser ophthalmoscopic images (**G**,**H**) clearly reveal the avascular vitreous veil (arrows), as well as retinal atrophy and pigment clumping. (**I**,**J**) Enlargement of the area within the box. Spectral-domain optical coherence tomography imaging of the macula (**K**) indicates outer retinal atrophy. (**L**,**M**): Pedigree of kindreds B and C. Arrows designate the probands, filled symbols represent affected individuals, clear symbols refer to unaffected individuals.

The proband (XDW894) in kindred A was a six-year-old boy adopted by his foster parents (foster father HSR863) who had poor vision and night blindness for two months. The best corrected visual acuity (BCVA) was 20/100 in his right eye and 20/50 in his left eye. The intraocular pressure (IOP) was normal. The anterior segments of both eyes were unremarkable on slit-lamp examination. Nevertheless, the fundus examination revealed osteocyte-like pigment clumping and chorioretinal atrophy (Figure 1A,B). Three months later, the patient was referred to our ophthalmologic center for sudden loss of vision. The fundus examination revealed rhegmatogenous retinal detachment of both eyes (Figure 1C,D). After pars plana vitrectomy (PPV) and silicone oil tamponade, the retina detachment receded. The retina remained attached at the six-month follow-up visit with BCVA 20/400 in his right eye and 20/200 in his left eye.

The proband (XDW789) of kindred B (Figure 1L) was a six-year-old girl who presented low vision in both of her eyes for two weeks. Her BCVA was FC/15 cm (finger counting with 15 cm) in the right eye and HM/30 cm (hand movement with 30 cm) in the left eye. The IOP was 6 mmHg in her right eye and 9 mmHg in her left eye. Pupillary synechiae and cataracts were found in her left eye (Figure 1E), while the anterior segment was normal in her right eye. Retinal detachment associated with retinal dialysis, combined with choroidal detachment, was noted in her right eye by fundus examination (Figure 1F). Due to the opacity of the lens of her left eye, ultrasonography was employed, which also revealed retinal and choroid detachment in her left eye. The proband’s mother (XDW790) was a 36-year-old who presented with severe cataracts of both eyes (data not shown).

In kindred C, the proband (XDW286) (Figure 1M) was a five-year-old girl who had been referred to our ophthalmologic center for exotropia. The BCVA was 20/400 in her right eye and 20/200 in her left eye. The intraocular pressure (IOP) was normal in both eyes. The axial length was 20.27 mm in the right eye and 19.55 mm in the left eye, with spherical equivalent (SE) as −2.75 diopters and −2.00 diopters, respectively. Dilated fundus examination revealed retina pigmentary degeneration and avascular vitreous veil in both eyes, a hallmark feature of Wagner syndrome (Figure 1G–J). Ectopic fovea was also detected, which may be associated with the 15° exotropia (pseudostrabismus) of the left eye. Optical coherence tomography (OCT) revealed the absence of the outer retinal layers under fovea (Figure 1K). Flash electroretinogram (ERG) was employed and showed a decrease in both rod and cone responses bilaterally, consistent with the extensive retinal abnormality affecting the photoreceptor layers on OCT (Supplementary Figure S1). The proband’s mother (XDW288) was a 30-year-old who had a history of exotropia, retinitis pigmentosa (RP) and cataracts for 5 years (Supplementary Figure S2). Her BCVA was 20/1000 in the right eye and 20/300 in the left eye, with SE as −14.00 diopters and −13.00 diopters, respectively. Osteocyte-like pigment clumping, chorioretinal atrophy and vitreous veils were identified in both of her eyes (Supplementary Figure S2) (Table 2). The dilated fundus examination of the proband’s sister (XDW289) revealed an optically empty vitreous, with a degree of chorioretinal atrophy at the retinal periphery (data not shown).

### 3.2. Genetic Findings

No genetic mutations were found in the three kindreds using WES. CNV analysis was then performed and identified the potential copy number variations. qPCR analysis was performed to confirm the CNV mutations. The relative quantity of the VCAN amplicon copy number ratio was close to 1 in the three kindreds (normal copy number ratio should be 2 because every exon has two copies in humans) by the qPCR analysis, confirming the deletion of VCAN in the heterozygous state. For the control genes (TTLL5-exon 14 and SPATA7-exon 6), a copy number ratio of around two was obtained, consistent with the two copies of each exon.

**Figure 2:**
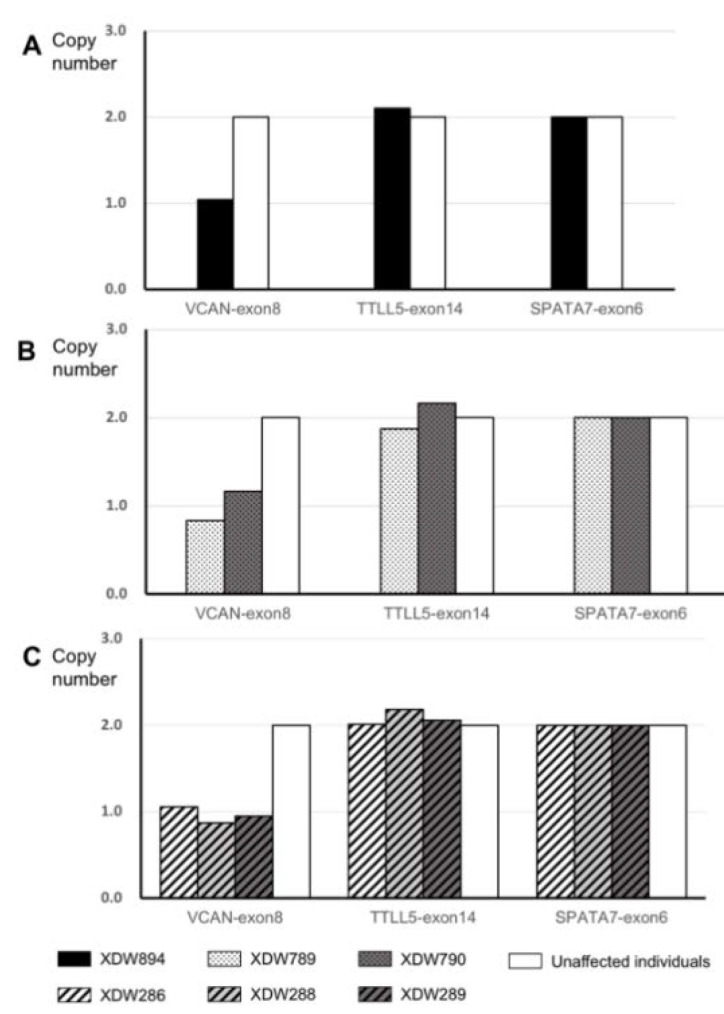
Molecular analysis of Wagner cases with VCAN exon 8 deletion. Representative histograms of qPCR of VCAN exon 8 from probands XDW894 (**A**), XDW789 (**B**) and XDW286 (**C**) compared to other family members of the same run. Unaffected individuals represent HSR863 in kindred A, XDW905 in kindred B and XDW287 in kindred C.

**Figure 3:**
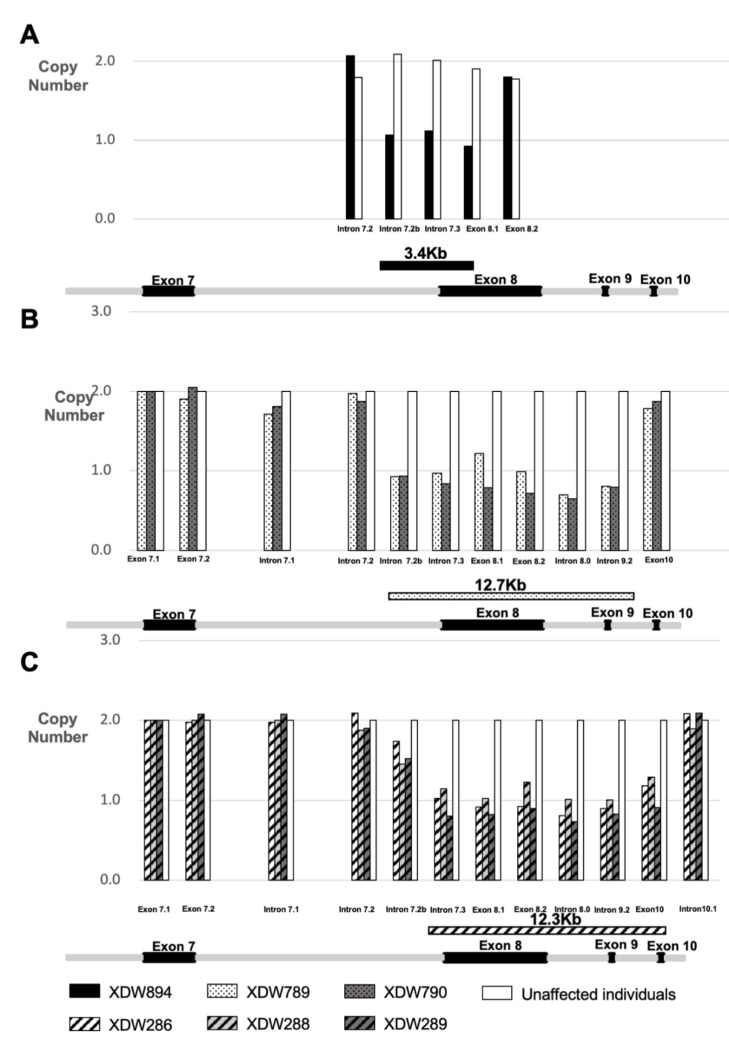
Confirmation and determination of the boundaries of each VCAN deletion using qPCR technology for three Wagner families. Each group of bars represents the copy number of a specific region of VCAN with primers specified in Supplementary Table S1. (**A**): CNV analysis of various positions of VCAN yielded a deletion from intron 7.2 b to exon 8.1 in kindred A. (**B**): CNV analysis of various positions of VCAN discovered a deletion from intron 7.2 b to intron 9.2 in kindred B. (**C**): Data analysis of qPCR indicated a deletion from intron 7.3 to exon 10 in kindred C. Unaffected individuals represent HSR863 in kindred A, XDW905 in kindred B and XDW287 in kindred C.

In kindred A, the copy number ratio of the exon 8 in VCAN for the proband (XDW894) reached 1.05, compared with the unaffected control samples (HSR863) (Figure 2A). In order to confirm the length of the deletions, real-time PCR with primers [12] (Supplementary Table S1) from exon 7 to exon 11 of VCAN was performed. CNV analysis of various positions of VCAN yielded a deletion of around 3.4 kb from intron 7.2 b to exon 8.1. The copy number ratios were 1.06, 1.11 and 0.92 in intron 7.2 b, intron 7.3 and exon 8.1, respectively, while a ratio of 2.07 and 1.80 was reached for intron 7.2 and exon 8.2 (Figure 3A).

In kindred B, CNV analysis revealed a copy number ratio of exon 8 in VCAN as 0.82 in the proband (XDW789) and 1.16 in the proband’s mother (XDW790) (Figure 2B) compared with the unaffected family member (XDW905). The CNV analysis of various positions of VCAN discovered a heterozygous deletion of around 12.7 kb from intron 7.2 b to intron 9.2. The same deletion was also detected in the proband’s mother (Figure 3B).

In kindred C, the copy number ratio of exon 8 in VCAN was 1.05 in the proband (XDW286), 0.95 in the proband’s sister (XDW289) and 0.87 in the proband’s mother (XDW288) compared with the unaffected family member (XDW287). The copy number ratios of the control genes (TTLL5-exon 14 and SPATA7-exon 6) were close to 2 (Figure 2C). Data analysis of the real-time PCR indicated a heterozygous deletion of around 12.3 kb from intron 7.3 to exon 10 of the VCAN gene (Figure 3C).

**Figure 4:**
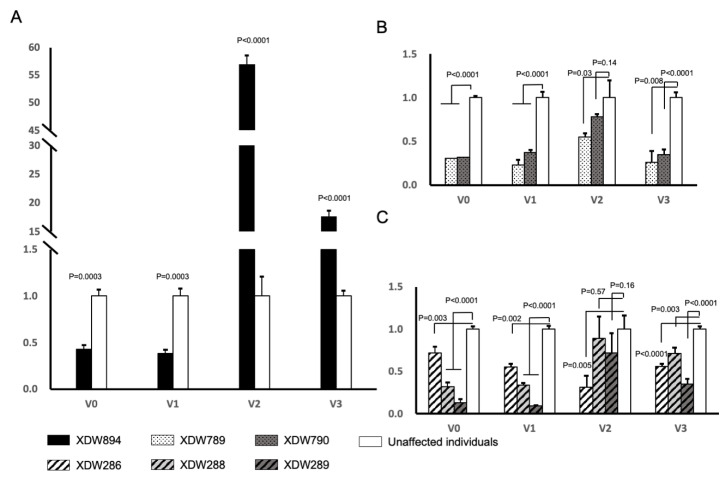
The relative expression level of V0, V1, V2 and V3 mRNA isoforms in three Wagner families by qPCR. (**A**): Relative mRNA level of XDW894 in pedigree A revealed a decrease in the quantitative ratio of V0 and V1, but an increase in V2 and V3 in pedigree A. (**B**,**C**): The quantitative mRNA ratio of four isoforms in affected individuals of pedigree B (panel B) and C (panel C) all decreased compared with the unaffected family member. Unaffected individuals represent HSR863 in kindred A, XDW905 in kindred B and XDW287 in kindred C.

In order to check if the VCAN deletion resulted in an imbalanced expression of VCAN transcript isoforms (Supplementary Figure S3), real-time PCR assays were carried out from the three kindreds. In kindred A, one exon–intron junction of exon 8 was removed, while the whole of exon 8 and at least part of exon 9 were deleted in kindred B and kindred C. As shown in Figure 4, the four isoforms of versican transcripts showed a substantial increase in the quantitative ratio of V2 and V3 isoforms (both *p* < 0.0001), with a decrease in the quantitative ratio of V0 and V1 isoforms (both *p* = 0.0003) in kindred A, consistent with previously reported studies [3,8,12,14]. In the other two kindreds, the VCAN deletion involved exon 9, as well as exon 8, which played a role in the transcription of isoforms V2 and V3. Different from kindred A, the quantitative ratio of the four isoforms all decreased, compared with the nonaffected family member with statistical significance in V0, V1 and V3.

## 4. Discussion

Since the VCAN gene mutation was proven to be associated with WD in 2005, only 23 families (Table 1) have been reported to present clear molecular evidence of WD. Of these, the majority of mutations are single base pair changes in the splice site of exon 8 of VCAN, which leads to imbalanced isoform ratios of versican, characterized by increased V2 and V3, but the haploinsufficiency of V0 and V1. In 2017, CNV mutation was first described by Burin-des-Roziers et al. in a Dutch family and a French family with two distinct deletions (10.5 and 3.4 kb) removing at least one exon–intron boundary of exon 8 [12]. During the same period, a Caucasian patient with a 11.7 kb deletion, including exon 8 of VCAN, was reported by Ankala et al. [11]. Considering the rarity of this disease, the spectrum of the genetic variability remains unclear. In the present study, we found three new CNV mutations of VCAN in three distinct WD families. It is worth mentioning that the 3.4 kb deletion (from intron 7.2 b to exon 8.1) found in kindred A in this study is different from the 3.4 kb deletion described by Burin-des-Roziers et al., which did not cover intron 7 but started from exon 8. Our report of two deletions containing both exons 8 and 9 of VCAN is the first report of the involvement of exon 9 in WD.

Our findings suggest that CNVs are important contributors to WD. Of the three clinically diagnosed WD patients with unidentified genetic mutations by WES, CNVs of VCAN were detected in all cases. To date, 15 studies, including 11 distinct single nucleotide mutations in 20 families and three distinct CNVs in three families, have been reported. Including the three previously reported WD families with CNV mutations, the rate of CNVs in reported WD families is 23.1% (6/26; three previously reported, three reported in this study). It may be the case that there are untested WD patients that can be attributed to CNVs. We suggest that CNVs are a common cause of VCAN gene mutations in WD. This result has important implications for the genetic diagnostic testing of patients with WD, suggesting that methods capable of detecting structural variations, such as whole-genome sequencing or WES accompanied with CNV analysis, should be used for WD patients. Bujakowska et al. also suggested that CNV mapping could provide a genetic diagnosis in around 7% cases with inherited retinal disease and should be included in genetic diagnostic testing [15]. 

Two families with deletions containing both exons 8 and 9 presented with characteristic ocular manifestations of WD. Normal intelligence, normal skeletal development, and normal facial features are noted in the patients. Different from kindred A, integral decreases in V0–V4 mRNA isoforms were measured in these patients, supporting the hypothesis that WD is caused by a deficiency in V0 and V1, but not compensatory increases in V2 and V3. This could explain the similar clinical features in patients with both exon 8 and 9 deletions in kindreds A and B. These results correct the impression that the VCAN mutation is confined to exon 8. Notably, the concept of pathogenic deficiency of versican isoforms is drawn from the mRNA levels in blood cells, which may not reflect the pathophysiology in the eye. Further studies in human eyes or appropriate animal models are needed to determine the physiological and pathological functions of the various isoforms of versican.

In conclusion, CNVs are important causes of WD. CNV analysis substantially increases the genetic diagnostic rate for WD. In addition to exon 8, the deletion of both exons 8 and 9 could give rise to WD.

## Figures and Tables

**Table 1:** Summary of published VCAN/versican splice site mutations and copy number variation (CNV) mutations of exon 8.

VCAN Gene Variant	DNA Nucleotide Change	Family Number	Reference
Intron 7 splice acceptor site	c.4004−1G > C	1	Kloeckener-Gruisse et al., 2013. [2]
	c.4004−1G > A	2	Mukhopadhyay, A. et al., 2006. [3]; JR, A., et al., 2018. [4]
	c.4004−1G > T	3	Chen X. et al., 2013. [5]; AS, T. et al., 2016. [6]
	c.4004−2A > G	2	Miyamoto, T. et al., 2005. [7]; PH, Tang. et al., 2019. [8]
	c.4004−2A > T	1	Brezin, A.P. et al., 2011. [9]
	c.4004−5T > C	4	Mukhopadhyay, A. et al., 2006. [3]
	c.4004−5T > A	1	Mukhopadhyay, A. et al., 2006. [3]
	c.4004−6T > A	1	Rothschild, P.R. et al., 2013b. [10]
Intron 8 splice donor site	c.9265 + 1G > A	3	Rothschild, P.R. et al., 2013. [11]; Kloeckener-Gruissem, B. et al., 2006. [12]; Meredith S.P. et al., 2007. [13]
	c.9265 + 1G > T	1	Ronan S.M. et al., 2009. [14]
	c.9265 + 2T > A	1	Kloeckener-Gruissem, B. et al., 2013. [2]
Copy number variation of exon 8	10.5 kb deletion	1	Burin-des-Roziers, C. et al., 2017. [15]
	3.4 kb deletion	1	Burin-des-Roziers, C. et al., 2017. [15]
	11.7 kb deletion	1	Ankala et al., 2018. [16]

**Table 2:** Summary of clinical characteristics of three Wagner families.

Pedigree/Patient ID Sex/Age	Clinical Diagnosis	Eye	BCAV	Refractive Status	Cataract	Empty Vitreous and Veils	Chorio-Retinal Atrophy	Retinal Pigmentary Changes	Retinal Detachment (Age, Years)	Other Ocular Abnormalities
A/XDW894 M/6	Wagner syndrome	OD	20/100	emmetropia	−	+	+	+	+(6 y)	
		OS	20/50	emmetropia	−	+	+	+	+(6 y)	
A/HSR863 M/30	Normal	OD	20/20	emmetropia	−	−	−	−	−	
		OS	20/20	emmetropia	−	−	−	−	−	
B/XDW789 F/6	Wagner syndrome	OD	FC/15cm	N/A	−	+	N/A	N/A	+(6 y)	
		OS	HM/30cm	N/A	+	+	N/A	N/A	+(6 y)	pupillary synechiae
B/XDW790 F/36	Wagner syndrome	OD	20/200	Mild myopia	+	+	N/A	N/A	−	
		OS	20/200	Mild myopia	+	+	N/A	N/A	−	
B/XDW905 M/38	Normal	OD	20/20	Mild myopia	−	−	−	−	−	
		OS	20/20	Mild myopia	−	−	−	−	−	
C/XDW286 F/5	Wagner syndrome	OD	20/400	mild myopia	−	+	+	+	−	
		OS	20/200	mild myopia	−	+	+	+	−	Ectopic fovea
C/XDW287 M/31	Normal	OD	20/20	emmetropia	−	−	−	−	−	
		OS	20/20	emmetropia	−	−	−	−	−	
C/XDW288 F/30	Wagner syndrome	OD	20/1000	high myopia	+	+	+	+	−	pseudostrabismus
		OS	20/300	mild myopia	+	+	+	+	−	exotropia
C/XDW289 F/3	Wagner syndrome	OD	N/A	mild myopia	−	+	+	+	−	
		OS	N/A	mild myopia	−	+	+	+	−	

N/A, not assessed.

## Supplementary Materials

The following are available online at https://www.mdpi.com/2073-4425/11/9/992/s1, Figure S1: Full-field electroretinograms of the proband (XDW286). Figure S2: Ocular findings of the proband’s mother (XDW288) in Kindred C. Figure S3: Diagram indicated the structures of the VCAN gene and the alternative transcript isoforms. Table S1: Listing of primers used for qPCR of genomic DNAs and qRT-PCR from mRNA samples of patients with exon 8 deletions.
