# Supplementary material for: Identification of Novel Copy Number Variations of VCAN Gene in Three Chinese Families with Wagner Disease
Source: Genes (Basel). 2020 Aug 25;11(9):992. doi: 10.3390/genes11090992 (PMC7564609; doi:10.3390/genes11090992)
Supplement: Supplementary file 1 [file genes-11-00992-s001.zip › genes-889558-supplementary/Supple Table.docx]

Supplemental Table

Listing of primers used for q-PCR of genomic DNAs and q-RT-PCR from mRNA samples of patients with exon 8 deletions.

| amplicon | Primer forward | Primer reverse |
| --- | --- | --- |
| Exon 7.1 | GCTAAAGAGGCTACAACCATCGATT | GTTTTGAACAGAAAGCCACAGTCC |
| Exon 7.2 | GGAACCTGGTGAAGAAACAACCA | GACCTCAGGCGCTTTCTACG |
| Intron 7.1 | CCTCCACTTCCCAATGCAGT | TGAGGATTGCGTTAGGCTGG |
| Intron 7.2 | CCAATCCCCCATGGCTACTG | GCCACATGCAAGTCACTGTG |
| Intron 7.2b | CAGTGAGCATTATGACCATCCA | AGCAATGAGAGACTCCTGGC |
| Intron 7.3 | TATGGGCAGGGTGGTGAGTA | TGCCCAAGACAAGGAGCTTT |
| Exon 8.1 | CACCCCATCTGTGCAGTACAT | TCTACAGAAACAACTGAGTCTCTTGAAG |
| Exon 8.1.2 | GCAGAATCAGTCACAGAGAGAGAT |  |
| Exon 8.1.3 | ACTAGCAGGATAATCACAGAAAGCTTTT |  |
| Exon 8.1.4 | ACCACTCTCCCACGTAGTCC |  |
| Exon 8.1.5 | AGTACCTACTTCAGTTCACATCAGTCA |  |
| Exon 8.2 | CAGAACAAACAATCTTTGATTCACAGAC | TACACAACTCTCCCTGAAGCTACT |
| Exon 8.3 | CCACGCTTTCTTCTTCTCCAGA | ATTCCAATGATCAGGCAACAGTAAAC |
| Intron 8.0 | TCATGGTCTCAGCATGGTGTC | TACCTTTTGTGCCACCAATG |
| Intron 8.1 |  | ATGTGGCTATGGCGTGTGTT |
| Intron 8.2 |  | TCAGGGTTTCAGGACTTAAGATTC |
| Intron 8.3 |  | TTCAGTGCACACTTTCGAAGA |
| Intron 8.4 |  | AATATGGTAGGACCTGATCGCTG |
| Intron 9.1 | CTTGGCTGAAAAGGTGCAGT | GTGAGGCCTGCTGAGTCTTC |
| Intron 9.2 | ACATGGCAGAACCTCACCTC | GCACTCAGCAAGCACCATAA |
| Intron 9.3 | TGGGAGAGGGAGACGTATTG | GCATCAGAGGGCTGGTAAAA |
| Exon 10 | CCTCACACTAAATGGAATTCTTTG | TTTTAGCAATTTTGGCCTCAA |
| Intron 10.1 | ATTTGCATTTTACGGCCAAC | ACATTGGTCTGGTCCCACAT |
| Intron 10.2 | AGTGCAGCTGTGCTCCTGT | TTGGTGATGACCGTGTCAGT |
| Intron 10.3 | CAGCTGGCTGCAACTAAACA | AGTTTCAATTTGGCCACCAA |
| Exon 11 | GCTCCTGCCTGTTTCTTCTC | ACGAGTTTCCAGGAACTTCA |
| V0 | GCACAAAATTTCACCCTGACATT | TGGATCTGTTTCTTCACTACAAGGTT |
| V1 | GGCTTCCCTCCCCCTGATA | TGGATCTGTTTCTTCACTACAAGGTT |
| V2 | GCACAAAATTTCACCCTGACATT | CGTTAAGGCACGGGTTCATT |
| V3 | GGCTTCCCTCCCCCTGATA | CGTTAAGGCACGGGTTCATT |
| SDHA | GCATTTCTACGACACCG | GTGCAATAGCGAGTGG |
