# Supplementary figures and images for: Identification of Novel Copy Number Variations of VCAN Gene in Three Chinese Families with Wagner Disease
Source: Genes (Basel). 2020 Aug 25;11(9):992. doi: 10.3390/genes11090992 (PMC7564609; doi:10.3390/genes11090992)

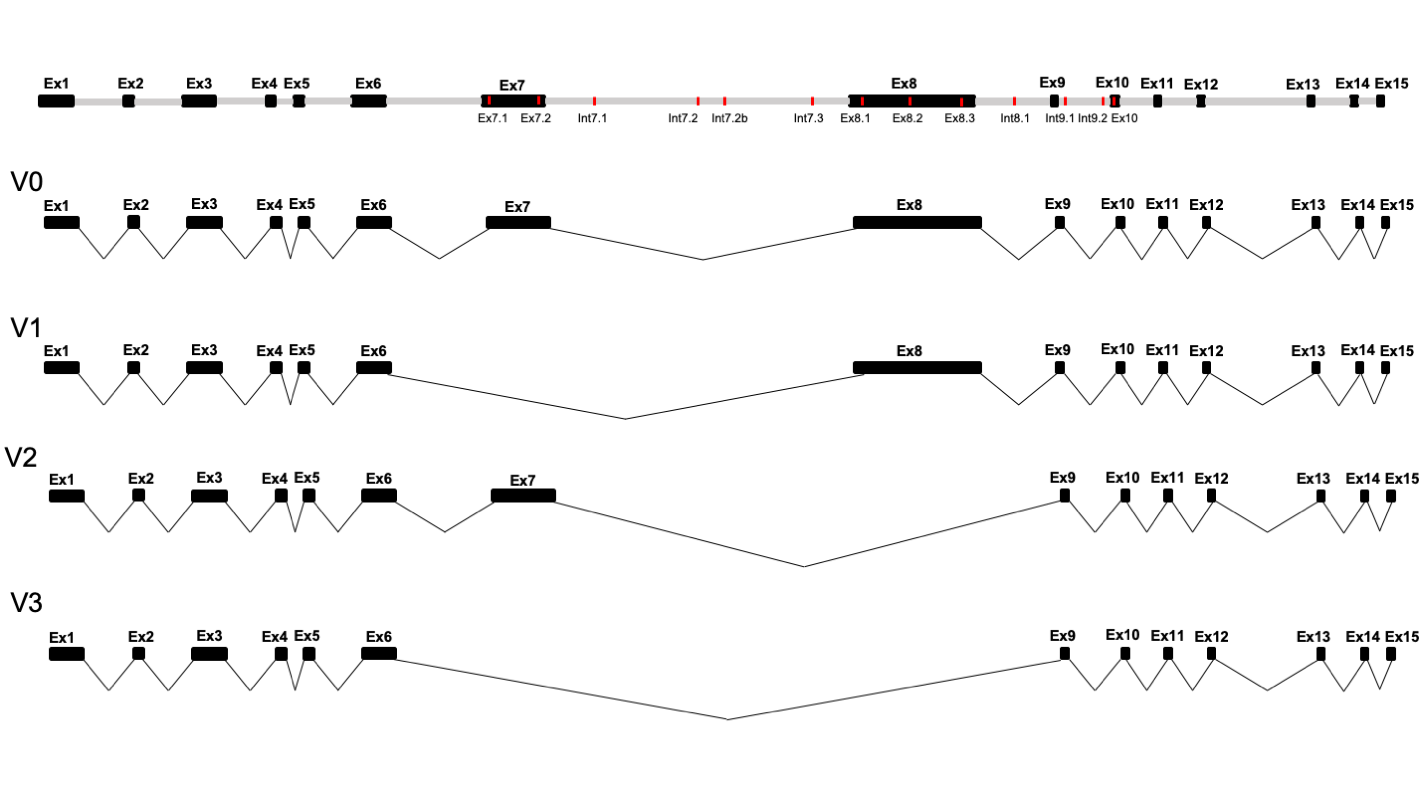

Supplement: Supplementary file 1 [file genes-11-00992-s001.zip › genes-889558-supplementary/Supplementary Figure 3 VCANgene+transcripts.tiff]

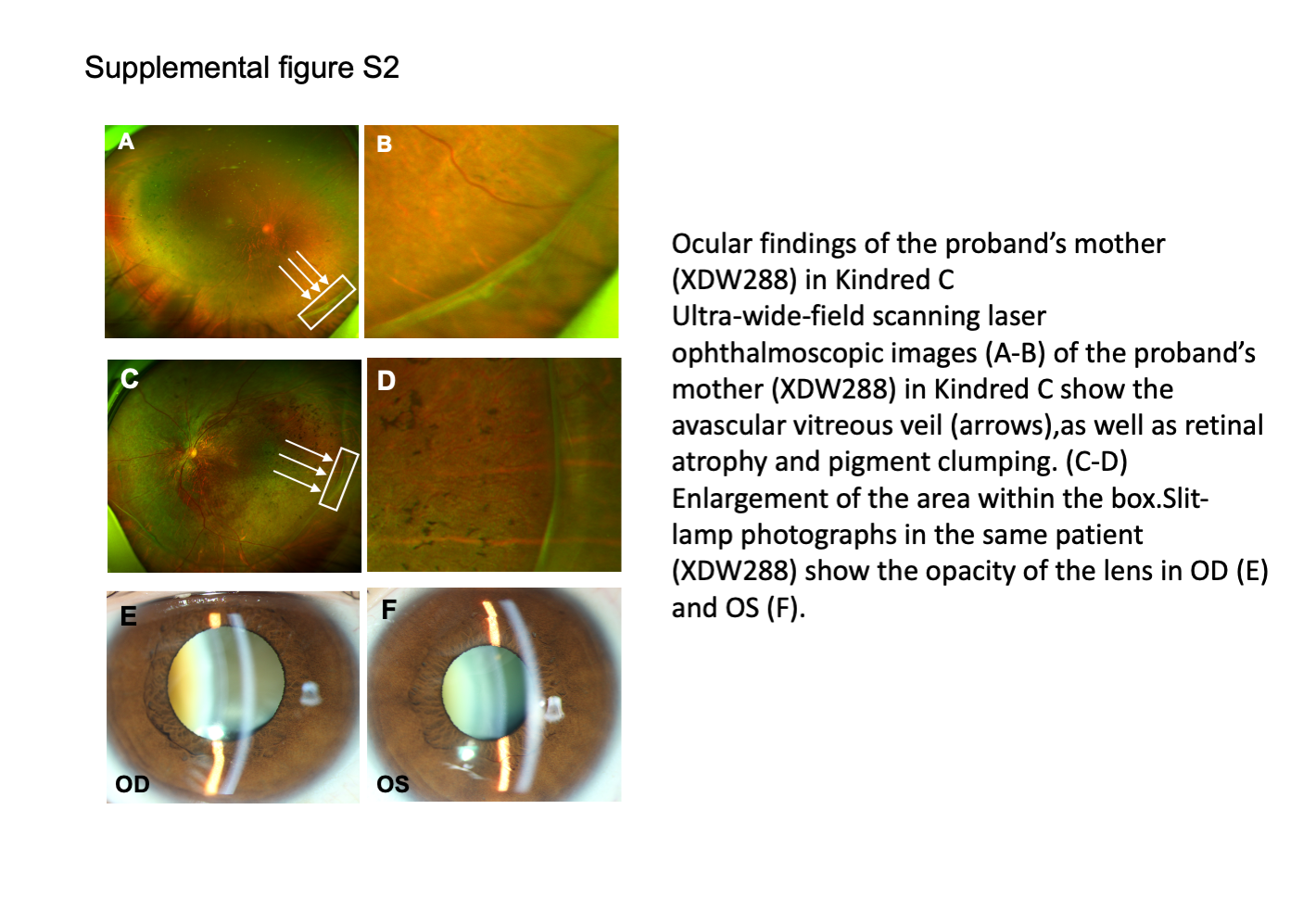

Supplement: Supplementary file 1 [file genes-11-00992-s001.zip › genes-889558-supplementary/Supplementary Figure 2.tiff]

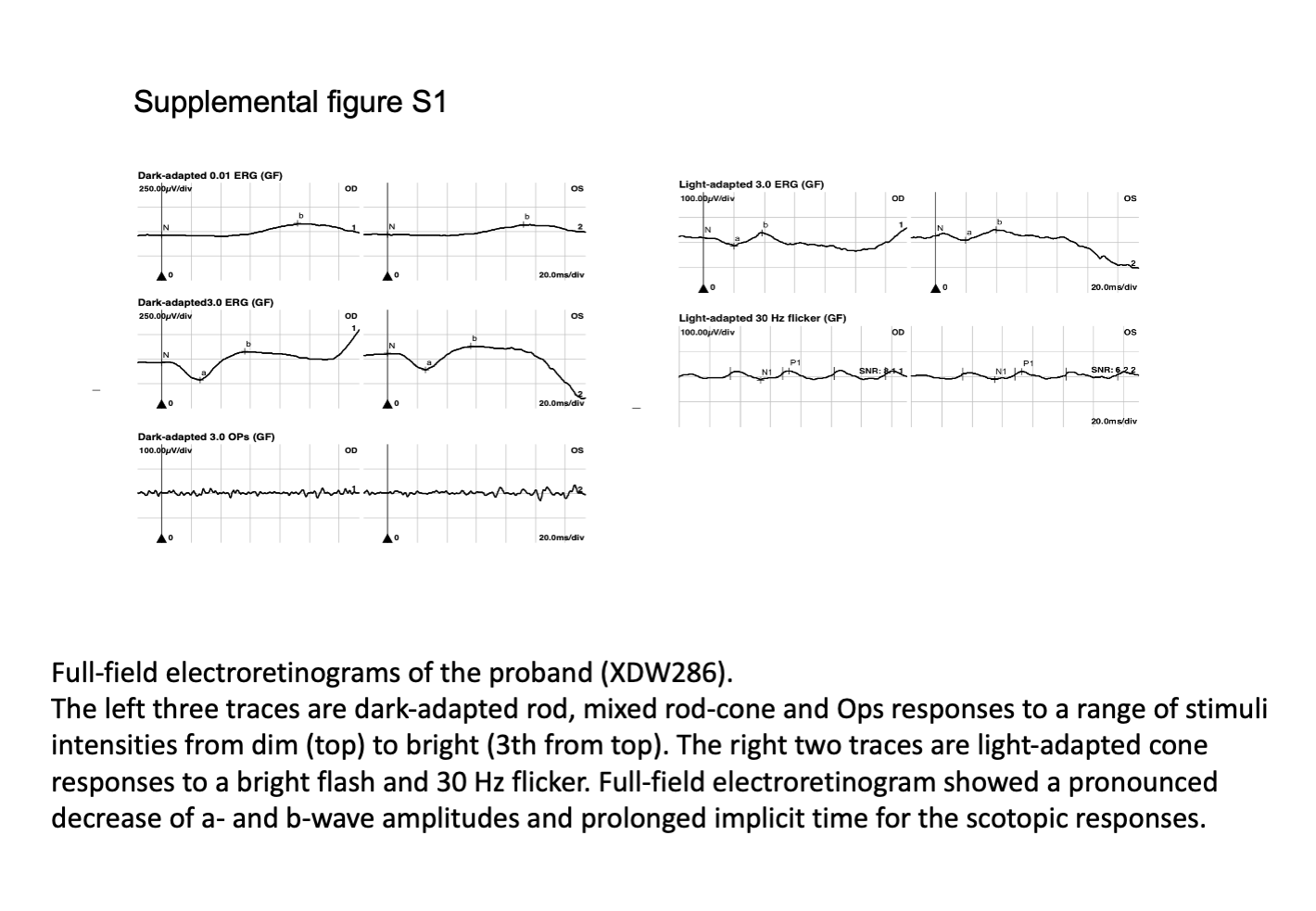

Supplement: Supplementary file 1 [file genes-11-00992-s001.zip › genes-889558-supplementary/Supplementary Figure 1.tiff]
